# Influence of Zinc Supplementation in Acute Diarrhea Differs by the Isolated Organism

**DOI:** 10.1155/2010/671587

**Published:** 2010-05-31

**Authors:** Archana B. Patel, Michael J. Dibley, Manju Mamtani, Neetu Badhoniya, Hemant Kulkarni

**Affiliations:** ^1^NGO, Lata Medical Research Foundation, Nagpur 440022, India; ^2^Department of Pediatrics, Indira Gandhi Government Medical College, Nagpur 440018, India; ^3^The Sydney School of Public Health, University of Sydney, 2006 Sydney, Australia; ^4^Department of Medicine/Infectious Diseases, University of Texas Health Science Center at San Antonio, San Antonio, TX, USA

## Abstract

Zinc supplementation is recommended in all acute diarrheas in children from developing countries. We aimed to assess whether zinc supplementation would be equally effective against all the common organisms associated with acute diarrheas. We used data on 801 children with acute diarrhea recruited in a randomized, double blind controlled trial (ISRCTN85071383) of zinc and copper supplementation. Using prespecified subgroup analyses, multidimensionality reduction analyses, tests of heterogeneity, and stepwise logistic regression for tests of interactions, we found that the influence of zinc on the risk of diarrhea for more than 3 days depended on the isolated organism—beneficial in *Klebsiella*, neutral in *Esherichia coli* and parasitic infections, and detrimental in rotavirus coinfections. Although we found similar results for the outcome of high stool volume, the results did not reach statistical significance. Our findings suggest that the current strategy of zinc supplementation in all cases of acute diarrheas in children may need appropriate fine tuning to optimize the therapeutic benefit based on the causative organism, but further studies need to confirm and extend our findings.

## 1. Introduction

In 2004, the World Health Organization (WHO) and the United Nations Children's Fund (UNICEF) recommended, in a joint statement, the use of zinc supplementation for the treatment of acute diarrhea in developing countries [1]. This recommendation was based on strong biological and epidemiological evidence which suggested that zinc supplementation can significantly reduce the overall duration of diarrhea and is also likely to reduce stool volume and frequency [2]. However, there exists significant heterogeneity in the effects of zinc on diarrhea-related outcomes observed across published randomized controlled trials [3–5]. Potential contributors to this heterogeneity are currently not fully understood.

There is some evidence to suggest that the beneficial effect of zinc may not be equivalent against the common causative organisms. Roy et al. [6] had first demonstrated that the extent to which mucosal permeability is affected in different diarrheas depends on the causative organisms—in general, diarrheas caused by invasive organisms show higher permeability. Second and consistent with this observation, Canani et al. [7] observed that zinc-induced promotion of ion absorption across the gut is evident in response to the ion secretion caused by *Vibrio cholerae* toxin but not the *Escherichia coli* heat-stable enterotoxin. Third, Surjawidjaja et al. [8] showed that although zinc sulphate can inhibit the growth of enteropathogens in vitro, the lethal dose required to kill 50% of the organisms (LD_50_) widely varies across the species of the causative organisms. Consequently it is possible that the overall beneficial effect of zinc supplementation observed in a trial may depend on the spectrum of the causative organisms within that study. The influence of zinc supplementation on diarrhea could thus be dependent on the organisms present in the gut. Using microbiological and clinical data from a three-arm randomized controlled trial of zinc supplementation, we therefore determined whether differential organisms can partially contribute to the effect of zinc. Due to limited resources, we were unable to conduct serotyping for pathogenic organisms causing diarrhea. In this report, therefore, we demonstrate the modulation of effect of zinc supplementation by bacterial isolates in the stool and rotavirus infection.

## 2. Patients and Methods

### 2.1. Study Subjects

This dataset comes from a double-blind, randomized, placebo-controlled clinical trial in children aged 6–59 months attending the Indira Gandhi Government Medical College and Hospital, in Nagpur, India with >3 unformed stools in the prior 24 hours; duration of diarrhea <72 hours; and ability to accept oral fluids or feeds. Details about the study subjects, the trial design, and its rationale are provided elsewhere [9]. Briefly, all children aged 6 months to 59 months attending study center with more than three unformed stools in the prior 24 hours with a total duration of diarrhea at recruitment of up to 72 hours and who were able to accept oral fluids were recruited in the study. The exclusion criteria were chronic or severe complicating illness, known positive HIV status, kwashiorkor, residing outside a radius of 30 km around the hospital, participating in another study, or already enrolled in this study. The trial is registered with the International Standard Randomized Controlled Trial register with the unique identifier ISRCTN85071383. The Ethics Committee of Indira Gandhi Government Medical College, Nagpur, and the Human Research Ethics Committee of the University of Newcastle, New South Wales, Australia (HREC Approval no: H-500-0203) approved the study protocol and the treatment effects monitoring committee monitored the trial for safety.

### 2.2. Study Protocol

Each recruited child was sequentially assigned to one of the following three treatment arms using a randomization protocol fixed a priori: placebo (Pl, *n* = 271) arm, zinc (Zn, *n* = 264) only arm, and zinc and copper (Zn + Cu, *n* = 273) arm. Participants in the Zn arm received the therapeutic dose 2 mg/kg/day of zinc while participants in the Zn + Cu arm received the same dose of zinc as well as 0.2 mg/kg/day of copper. Microbiological investigations were conducted with a sterile container having a plastic spoon attached to the inside of the screw cap for stool collection. The fecal sample first underwent a naked eye examination for consistency, presence of mucous and blood. In the laboratory, the sample underwent gross and microscopic examination of wet and iodine preparations. Kenyon's method of acid fast staining for parasitic cyst was also done. For bacterial isolation sample was inoculated on sheep blood agar, MacConkey Bile Salt Agar, selenite F broth, Skirrow's campylobacter medium, alkaline peptone water, and thiosulphate citrate bile salts agar. The bacterial isolates were identified by standard bacteriological techniques [10]. Enzyme-linked immunosorbent assay (ELISA; Premier Rotaclone, Meridian Bioscience, Inc, Cincinnati, OH) was used for rotavirus antigen detection.

### 2.3. Outcomes and Predictors

In this study we examined whether zinc supplementation either alone or in combination with copper was associated with improvement in two diarrhea-related outcomes—the likelihood of continued diarrhea beyond 3 days since initiation of therapy, and the volume of stool collected during hospital stay. This latter outcome was divided into two categories based on the median stool volume in the study subjects. This study was conducted as a set of prespecified subgroup analyses that specifically aimed to tease out possible heterogeneity of zinc supplementation effects across the spectrum of the organisms isolated from stools.

### 2.4. Statistical Analyses

We followed the existing guidelines for reporting subgroups analyses from randomized controlled trials [11, 12]. Recognizing that the interpretations and recommendations based on subgroups analyses heavily depend on a critical and careful statistical analysis, we conducted our analyses in two steps. 

In the first step, we attempted to establish that our analyses will have sufficient statistical power while addressing the issues of multiple comparisons and of treatment (zinc) × modifier (isolated organisms) interactions. For this we used the following three complementary analytical techniques. (a) The first is the method of multifactor dimensionality reduction (MDR) [13] to examine the likely interactions among isolated organisms and zinc supplementation for the two diarrhea related organisms. MDR is a nonparametric, exploratory approach that identifies and characterizes the potential interactions in a large number of discrete predictors with a binary outcome and is commonly used to identify gene-gene interactions. (b) We next looked for significant bivariate interaction of each major isolated organism with zinc supplementation for both the outcomes. For this, we used the interaction contrast statistic (Δ) described by Gönen [14]. We tested the statistical significance of this interaction using the Breslow-Day test for heterogeneity and also determined the statistical power of our study to make inferences about these interactions. To estimate the power we made use of the *θ* statistic [14]. This was estimated using the arcsine transformation as 2sin^−1^ (*p* ^1/2^)where *p* is proportion of subjects developing the outcome of interest for a given combination of the therapy received and the isolated organism. The *θ* statistic has a standard normal distribution with variance 1/*n*, where *n* is the number of observations. (c) We next conducted stepwise multiple logistic regression analysis [15] for each outcome where the primary objective was to arrive at a reduced list of significant interactions based on the organisms identified in step (b). For stepwise regression we used the backward elimination strategy with a conventional probability retention criterion of 0.2. In the full model, we considered the main effects and all interactions (bivariate as well as higher-order) among the therapy and diarrheal isolates. One cardinal issue in subgroup analyses is the multiplicity of comparisons. To address this, we counted the total number of comparisons and reported the likelihood of false positive identification of significant interactions.

In the second step of the analysis, we aimed to test the robustness of our findings in the light of possible correlations with other predictors of zinc effectiveness. In this step, we used multivariate unconditional logistic regression modeling. In these multivariate models we adjusted for the following covariates: age, gender, presence of stunting (weight-for-age z-score <−2), presence of wasting (length-for-age z-score <−2), wealth index score, hand washing score, baseline plasma zinc level, baseline plasma copper level, and baseline hemoglobin level. Statistical significance was examined at a type I error rate of 0.05. 

MDR analysis used the MDR software (http://sourceforge.net/projects/mdr/). All other analyses were conducted using Stata 10.2 (Stata Corp, College Station, TX) software package.

## 3. Results

### 3.1. Description of Outcomes and Predictors

Of the total 808 study subjects, microbiological data was available for 801 (99.1%) children. From these 801 children a total of 913 organisms were isolated—548 (68.4%) children had a single organism isolated, 195 (24.3%) children had mixed isolates with at least 2 organisms, while no organisms could be isolated in 58 (7.3%) children. In the order of commonness the following organisms were isolated in the study subjects: *E. coli * 451 (56.3%), *Klebsiella spp *276 (34.5%), rotavirus 169 (21.1%), parasitic ova and cysts in stool 121 (15.1%), *Shigella spp  * 8 (1%), *Salmonella spp * 5 (0.6%), and *Campylobacter jejuni *2 (0.2%). Interestingly, of the 169 children with rotavirus infection, *E. coli-*rotavirus combination was observed in 100. At baseline, children with weight for age <−2 z-score were 19%, 57.5%, and 32.7% in those with rotavirus infection, *E.coli* and *Klebsiella* isolates in the stool samples. Correspondingly the proportions with serum zinc <60 *μ*g/dl were 25.2%, 56.4%, and 35%, respectively. Adherence to supplementations was similar in the three groups at 92.2%, 90.4%, and 88%, respectively. Three hundred and six (38.2%) children had diarrhea that extended beyond 72 hours after initiation of therapy. The median stool volume for all the study subjects was 685 ml. Based on this cut-off we dichotomized the study children in to those who had low stool volume (<685 ml) and those who had high stool volume (≥685 ml).

### 3.2. Interactive Effects among Isolated Organisms and Zinc Supplementation

**Figure 1:**
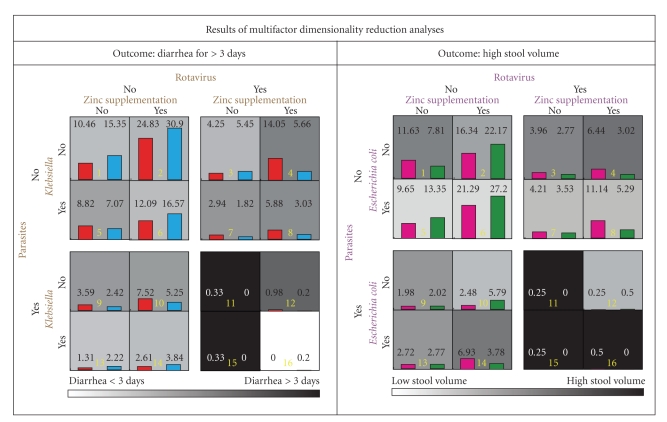
Investigation into the interaction of isolated organisms with the effect of zinc on two diarrhea-related outcomes—risk of continued diarrhea for 3 or more days (left column) and high stool volume (right column) based on the results from multifactor dimensionality reduction (MDR) analyses. Results show the proportion of subjects with (red and magenta bars) and without (blue and green bars) the indicated outcome for a given combination of zinc supplementation and isolated organisms. The background of each cell representing a specific combination is gradient, coded as shown in the key at the bottom of the MDR grid. Numbers at the top of the bars are proportions and numbers in yellow are cell identifiers.

We first conducted MDR analyses to identify and characterize potential interactions among zinc supplementation and four major isolates: *E. coli*, *Klebsiella*, rotavirus, and parasites. Our results indicated (Figure 1) that for the outcome of diarrhea >3 days *E. coli* was not found to be contributory to any interaction while *Klebsiella* was noncontributory to the outcome of high stool volume. Presence of rotavirus was associated with a higher proportion of children with diarrhea >3 days especially when they received zinc supplementation as indicated by taller red bars in cells 4, 8, and 12 in Figure 1. On the other hand, when *Klebsiella* but not rotavirus was isolated from the stools, zinc supplementation appeared to be associated with a reduced likelihood of diarrhea >3 days (compare the red and blue bars in cells 6, 13, and 14 of Figure 1). Although MDR included parasites as another potential source of interactions for the outcome of diarrhea >3 days, its role did not appear to be definitive. Using this set interactions identified by MDR, its odds ratio for accurate prediction of the outcome was 2.34 (95% CI 1.73–3.17, *P* = 2.6 × 10^−8^) with an overall accuracy of 63.1%. For the outcome of high stool volume, MDR analysis again identified presence of rotavirus as a potential deterrent to the beneficial effect of zinc (magenta bars in cells 4, 8, 12, and 16 in Figure 1). In general, presence of *E. coli * in the stool was associated with an increased risk of high stool volume (magenta bars in cells 7, 8, and 13–16 in Figure 1). Again the role of parasites, although identified by MDR as a possible dimension, was equivocal. For this outcome, the OR for correct prediction based on MDR results was 2.33 (95% 1.73–3.14, *P* = 1.7 × 10^−8^) with an overall accuracy of 59.4%.

**Figure 2:**
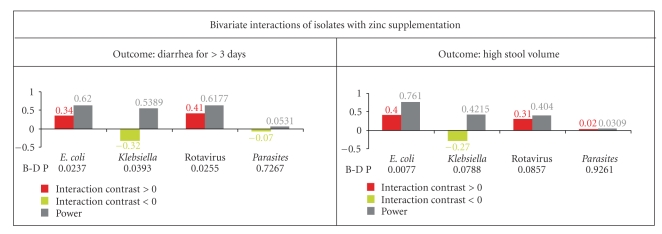
Statistical evaluation of the bivariate interactions between zinc supplementation and each of the four major isolates for two diarrhea-related outcomes—risk of continued diarrhea for 3 or more days (left column) and high stool volume (right column). The interaction was quantified using the interaction contrast statistic [14], the significance was tested using the Breslow-Day (B-D P) test for heterogeneity, and statistical power of interpretation in the present study was determined using the *θ* statistic described by Gönen [14]. Interaction contrast exceeding or below zero indicates a detrimental or protective interaction, respectively.

We next examined the bivariate interactions between each of the major isolates and zinc supplementation for both the outcomes (Figure 2). For diarrhea >3 days, the interaction constant was positive for *E. coli* and rotavirus, significantly negative for *Klebsiella,* and uninformative for parasites. This indicated that zinc supplementation may provide detrimental results in the presence of *E. coli* and rotavirus but may be associated with a shorter period of diarrhea in the case of *Klebsiella* isolates. The Breslow-Day test also identified *E. coli*, rotavirus, and *Klebsiella* to be interacting significantly with zinc supplementation for this outcome. As shown in Figure 2 (grey bars) our study had moderate to sufficient power to detect significant interactions. Although similar directionality for interactions was also observed for the outcome of high stool volume, statistical significance as well as sufficient power was only achieved for the *E. coli* isolates. Since parasites did not demonstrate any significant interaction with zinc supplementation and had negligible statistical power for either of the outcomes, we omitted this source of interaction from all further analyses. 

**Figure 3:**
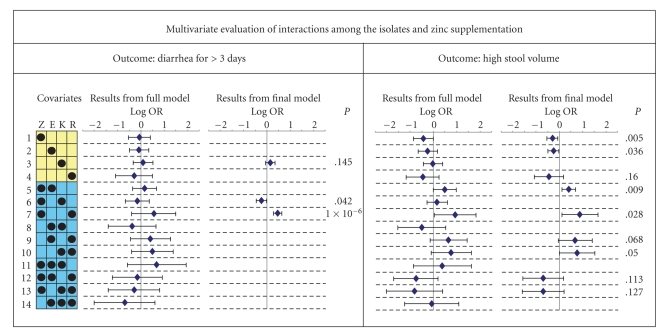
Multivariate association of main and interactive effects of zinc supplementation and diarrheal isolates using stepwise logistic regression models for two diarrhea-related outcomes—risk of continued diarrhea for 3 or more days (left column) and high stool volume (right column). In this grid, yellow background indicates the main effects, black circle indicates the included covariate, the blue background indicates interactive effects, and black circles in the blue part of the grid indicate the interactions among the circled covariates. Z: zinc supplementation; E: *Escherichia* *coli*; K: *Klebsiella*; and R: rotavirus. Diamonds and error bars indicate the point and 95% confidence interval estimates for the log odds ratio (Log OR). Results are from full model (on the left) and final model (on the right) from the stepwise logistic regression analyses. The full model included all the 14 covariates indicated in the grid while the stepwise regression analyses used a backward elimination strategy with a probability retention criterion of 0.2. Numbers at the right-hand side in the final model plots are significance values for the covariates that were retained in the final model.

Lastly, we considered the importance of the interactions in a multivariate context. We studied four main effects (zinc, *E. coli*, *Klebsiella,* and, rotavirus), six bivariate interactions (listed as covariates 5 through 10 in Figure 3), and four three-way interactions (covariates 11 through 14 in Figure 3). To control for multiple comparisons in this step of the analysis we employed the method of stepwise logistic regression with a conventional retention criterion of 0.2. In the final models, we observed that for the outcome of diarrhea >3 days zinc supplementation provided two statistically significant and directionally opposite interactions: with rotavirus and (OR > 1) and with *Klebsiella *(OR < 1). In contrast, for the outcome of high stool volume there were two synergistic and significant interactions of zinc supplementation: with *E. coli* and with rotavirus (both OR > 1). Thus, there were four significant interaction terms that involved zinc supplementation in our stepwise multivariate regression procedure. As shown in Figure 3, there were a total of 10 interaction terms in the full model for each outcome, and thus, a total of 20 interactions were considered in the analysis. At a type I error rate of 0.05, this would mean that one of the significant interactions detected in our study is likely to be a false positive.

### 3.3. Robustness of Treatment Heterogeneity: Multivariate Models

We next set out to investigate whether the significant interactions observed held the significance in the face of several other potential contributors to the two outcomes. Consistent with our previous observations [9], we found that zinc supplementation either alone or in combination with copper supplementation did not influence either of the study outcomes after adjusting for baseline covariates (rows titled “Overall” in Tables 1 and 2). We conducted a series of multivariate logistic regression models that included the aforementioned covariates.

For the outcome of continued diarrhea >3 days, we observed that zinc supplementation had a strikingly protective effect when *Klebsiella* was isolated, no effect when single *E. coli* or intestinal parasites were isolated, but a high risk of the outcome when rotavirus was isolated and an even higher likelihood (3.39 times) of prolonged diarrhea when *E. coli* were isolated along with rotavirus. Moreover, the protective effect in the context of *Klebsiella* isolates was nullified when rotavirus isolates were simultaneously found. Notably, we observed the protective effects of zinc supplementation when *Klebsiella *was isolated and the detrimental effects of zinc supplementation in *E. coli*-rotavirus infections were boosted by the addition of copper. We found similar results when we used other cut-points for dichotomizing the diarrheal duration like 5 days and 7 days (data not shown). Thus, the influence of zinc supplementation on the proportion of subjects with prolonged diarrhea was substantially modulated by the micro-organisms isolated. For the outcome of high stool volume we observed similar effects; however, in most instances the observations did not reach statistical significance (Table 2).

## 4. Discussion

There are four-key findings of this study. First, the beneficial effect of zinc supplementation in acute diarrhea was not equal against all organisms isolated from stools. Of special concern, however, was our observation that zinc may actually increase the risk of prolonged diarrhea in the presence of *E. coli*-rotavirus (prevalence of 12% in this study). Prescreening for rotavirus reactivity may enhance the utility of zinc supplementation by restricting it to subjects not reactive to rotavirus. It is interesting to note that two studies in young infants in four countries (Bangladesh, Ethiopia, India, and Pakistan) showed no effect of zinc supplementation on duration of acute diarrhea. Rotavirus is known to be the commonest causative organism in breast fed young infants, and although the authors acknowledge that it could be the reason for failure of zinc effect, it was not assessed in any of these studies [16, 17]. The only other randomized controlled trial [18] that has conducted a subgroup analysis based on the presence or absence of rotavirus in diarrhea concurs with our finding that the beneficial effect of zinc could not be observed in rotavirus-infected children. Diarrhea in rotavirus infection may be caused by several mechanisms, namely, enterocyte destruction, villus ischaemia, activation of enteric nervous system by release of vasoactive agents, and intestinal secretion by intracellular and extracellular action of nonstructural protein (NSP4) which stimulates Ca^++^ dependant cell permeability [19]. The action of zinc supplementation on these mechanisms is currently unknown. Our findings suggest that in rotavirus infected children zinc should be used cautiously. Our results also gain operational importance since in many situations it may not be feasible to conduct microbiological studies in deciding the value of zinc supplementation in children with acute diarrhea.

Second, in children from whom the commonest organism (*E. coli*) in the present study was isolated, zinc supplementation showed no significant association with the length of diarrhea or stool volume. This is despite the fact that this group had the highest proportion of wasted children, had serum zinc <60 *μ*g/dl, and most could thus be expected to benefit from zinc supplementation. Including this study, two other trials [20, 21]—both from India—had found that *E. coli* was the commonest stool isolate in children with diarrhea. One of these studies [21] found no significant impact of zinc supplementation on diarrheal duration while the other study [20] found a significant benefit but had used a high dose (40 mg/Kg/d) of elemental zinc. This finding is interesting in the light of the observation [8] that a higher concentration of zinc sulphate is needed in vitro to inhibit the enteropathogenic strains of *E. coli*. Together these observations suggest that the possibility of higher dose zinc supplementation should be considered when *E. coli* is the probable cause of diarrhea. Our findings along with those of Surjawidjaja et al. [8], Dutta et al. [20], and Sachdev et al. [21] also raise an interesting possibility that an optimum dose of zinc may be beneficial against most of the causative organisms and that a possible future direction to take would be to design studies focused on the dose-response to zinc supplementation. It should be remembered however that in vitro, ex vivo, or in vivo benefit of zinc supplementation in rotavirus diarrheas is currently unstudied and unknown.

Third, although mechanistically unclear, our results suggest that *Klebsiella*-associated diarrheas may be more responsive to zinc supplementation. *Klebsiella* enterotoxins cause reduced Na absorption and net Cl^−^ secretion in rabbit ileum by increasing cGMP. Although the addition of zinc does not affect cGMP-mediated ion secretion, zinc may still have a protective effect that is associated with its action on basal ion transport [7]. It is well recognized that the enteroaggregative patterns exhibited by *Klebsiella spp* and their heat-stable enterotoxins are structurally and functionally distinct from those of *E. coli* and chiefly induce diarrhea by chloride ion depletion [22, 23]. Since it has been shown that zinc can specifically inhibit the luminal secretion of the chloride ion [7, 24], it is likely that the *Klebsiella*-induced diarrhea is amenable to zinc supplementation. Considering the similarity in the mechanism of diarrhea causation it is conceivable that zinc supplementation may also be very useful in cholera—a premise that is strongly supported by in vitro and epidemiological observations [7, 25]. Finally, our results shown in Table 2 indicated that the significant interactions observed for the outcome of high stool volume may likely have been false and could have originated from the complex correlation structure among the covariates that better predicted the outcome. This reiterates the general view that zinc supplementation has a more significant effect on the diarrheal duration rather than on the volume of stool. 

We believe that the limitations of this study must be recognized before a generalization of the results can be undertaken. First, we did not have the resources to serotype the organisms isolated in the stools. Consequently, it cannot be presumed that the isolated organisms would necessarily indicate a causal association with the diarrhea episode. Nevertheless we could demonstrate a differential response in rotavirus infection and in the presence of different bacterial organisms in the gut regardless of the fact that some may not have been pathogenic. Second, this study only had statistical power in excess of 60% which can be considered as sufficient but not very high. Thus, replication and substantiation of our findings in larger and more statistically powerful studies is needed. Third, our findings are essentially epidemiological. Whether they readily translate to bedside management cannot be forecast in the absence of more direct mechanistic studies. This study suggests that more research is needed to understand the effect of zinc supplementation on acute diarrhea due to different causative pathogens. Therefore we suggest that zinc efficacy trials should now include a more complete assessment of the causative organisms at baseline. Fourth, microbiological prescreening prior to zinc supplementation in resource-limited countries is unlikely to be feasible. Thus, future studies should address this important area of interventional feasibility.

## 5. Conclusion

There is now a growing recognition that the beneficial effects of zinc supplementation may not be universal [9, 26]. Although we did not have data on further typing of the isolates in this study, our findings suggest that the universal strategy of zinc supplementation regardless of the organism isolated in the stool may be an oversimplification. If our results are indeed pointing towards a true differential benefit of zinc supplementation by the causative organisms, then more care will be required in recommending an optimum dose of zinc that is most beneficial as it will depend on the differential LD_50_ values [6] and the relative prevalence of the microbes included in the causative spectrum of acute diarrhea.

## Figures and Tables

**Table 1:** Influence of zinc supplementation on risk of diarrhea >3 days in different subgroups based on the isolated organisms.^a^

Subgroup	*N*	Zn verses Pl			Zn + Cu verses Pl			Any zinc^b^ verses Pl		
		OR^c^	95% CI	*P*	OR	95% CI	*P*	OR	95% CI	*P*
All children										

Overall	798	1.16	0.81–1.67	.421	1.05	0.73–1.50	.808	1.10	0.80–1.51	.547
*E. coli* infection	449	1.59	0.96–2.61	.069	1.42	0.97–2.33	.164	1.50	0.97–2.32	.068
*E. coli* single infection	273	1.32	0.68–2.57	.413	1.16	0.59–2.26	.667	1.24	0.69–2.22	.477
*Klebsiella* infection	275	0.73	0.39–1.36	.322	0.58	0.31–1.09	.092	0.65	0.38–1.12	.121
*Klebsiella* single infection	170	0.46	0.20–1.05	.065	0.43	0.18–0.98	.045	0.44	0.22–0.90	.025
Rotavirus infection	169	2.30	0.98–5.39	.054	3.00	1.31–6.87	.009	2.65	1.28–5.48	.009
*E. coli* + rotavirus mixed infection	100	2.43	0.71–8.33	.159	4.54	1.33–15.5	.016	3.39	1.13–10.1	.029
*Klebsiella* + rotavirus mixed infection	53	2.29	0.36–14.6	.381	1.50	0.33–6.91	.600	1.72	0.42–7.05	.444

Children with RBCs in stool^d^										

Overall	334	1.33	0.74–2.40	.341	1.04	0.58–1.88	.879	1.18	0.71–1.97	.537
*E. coli* infection	193	2.20	0.99–4.91	.054	1.81	0.83–3.91	.135	1.98	0.98–3.98	.056
*E. coli* single infection	121	2.21	0.69–7.10	.183	1.44	0.47–4.51	.522	1.76	0.62–4.91	.282
*Klebsiella* infection	109	0.51	0.15–1.65	.260	0.26	0.08–0.91	.035	0.37	0.13–1.08	.069
*Klebsiella* single infection	64	0.37	0.07–2.12	.262	0.23	0.04–1.23	.085	0.28	0.06–1.27	.099

^a^All the results are from multivariate logistic regression analyses which adjusted for effects of age, gender, presence of stunting (weight-for-age z-score <−2), presence of wasting (length-for-age z-score <−2), wealth index score, hand washing score, baseline plasma zinc level, baseline plasma copper level, and baseline hemoglobin level.

^b^Children receiving zinc alone or zinc and copper compared to those receiving placebo.

^c^OR: odds ratio; CI: confidence interval; *N*: number of subjects with complete covariate information; Zn: zinc, Zn + Cu: zinc and copper; Pl: placebo.

^d^There were only 28 children with Rotavirus isolates who had RBCs in stool. Due to this small number, analyses separately for the Rotavirus group have not been carried out.

**Table 2:** Influence of zinc supplementation on the outcome of high stool volume in different subgroups based on the isolated organisms.^a^

Subgroup	*N*	Zn verses Pl			Zn + Cu verses Pl			Any zinc^b^ verses Pl		
		OR^c^	95% CI	*P*	OR	95% CI	*P*	OR	95% CI	*P*
Overall	798	0.88	0.62–1.26	.496	0.88	0.62–1.25	.472	0.88	0.65–1.20	.418
*E. coli* infection	449	1.17	0.72–1.89	.519	1.41	0.87–2.26	.159	1.29	0.85–1.95	.236
*E. coli* single infection	273	0.97	0.52–1.81	.922	1.20	0.65–2.22	.560	1.08	0.63–1.86	.776
*Klebsiella* infection	275	0.67	0.36–1.24	.204	0.55	0.30–1.03	.062	0.61	0.36–1.04	.071
*Klebsiella* single infection	170	0.70	0.31–1.58	.395	0.48	0.21–1.08	.077	0.58	0.29–1.18	.131
Rotavirus infection	169	1.44	0.62–3.39	.398	2.31	0.99–5.39	.052	1.85	0.89–3.84	.097
*E. coli* + rotavirus mixed infection	100	1.19	0.35–4.00	.781	2.44	0.72–8.27	.151	1.74	0.59–5.09	.313
*Klebsiella* + rotavirus mixed infection	53	0.92	0.14–6.08	.930	2.09	0.37–11.9	.408	1.51	0.32–7.07	.601

^a^All the results are from multivariate logistic regression analyses which adjusted for effects of age, gender, presence of stunting (weight-for-age z-score <−2), presence of wasting (length-for-age z-score <−2), wealth index score, hand washing score, baseline plasma zinc level, baseline plasma copper level, and baseline hemoglobin level.

^b^Children receiving zinc alone or zinc and copper compared to those receiving placebo.

^c^OR: odds ratio; CI: confidence interval; *N*: number of subjects with complete covariate information; Zn: zinc, Zn + Cu: zinc and copper; Pl: placebo.
